# Presence of SARS‐CoV‐2 virus in wastewater in the Kingdom of Bahrain during the COVID‐19 pandemic

**DOI:** 10.1111/irv.13194

**Published:** 2023-11-13

**Authors:** Afaf Merza Mohamed, Ebrahim Matar, Hasan M. Isa, Ahmed K. Moosa, Wafa Fawzi Hasan, Amjad Ghanem Mohamed, Adel Salman Al Sayyad, Maryam Y. Sanad, Maryam Alhajeri, Najat Abu Alfatah, Qasim M. Alaraibi

**Affiliations:** ^1^ Public Health Directorate Ministry of Health Manama Bahrain; ^2^ Eastern Health Cluster Dammam Saudi Arabia; ^3^ Pediatric Department, Salmaniya Medical Complex Arabian Gulf University Manama Bahrain; ^4^ Mansoura University Mansoura Egypt; ^5^ Family Medicine, Epidemiology & Public Health, Disease Control Section, Ministry of Health. Family and Community Medicine, CMMS AGU Manama Bahrain; ^6^ Food and Water Microbiological Analysis, Public Health Directorate Ministry of Health Manama Bahrain; ^7^ Ministry of Health Manama Bahrain; ^8^ Al Malki Hospital Riffa Bahrain; ^9^ Ministry of Agriculture and Municipality Manama Bahrain

**Keywords:** Bahrain, COVID‐19, SARS‐CoV‐2, surveillance, wastewater

## Abstract

**Background:**

Several countries, including Bahrain, used wastewater surveillance for disease activity monitoring. This study aimed to determine the presence of SARS‐CoV‐2 in untreated wastewater and to correlate it with the disease spread.

**Methods:**

A retrospective review was conducted for all wastewater samples tested for SARS‐CoV‐2 in public health laboratories from November 2020 to October 2022. Samples were collected weekly between February and October 2022 from different areas across Bahrain. Real‐time polymerase chain reaction was used to test for the presence of SARS‐CoV‐2 in wastewater, and the results were correlated with the number of COVID‐19 cases in the same area.

**Results:**

Of 387 wastewater samples, 103 (26.6%) samples tested positive for SARS‐CoV‐2. In late 2020, of 42 samples collected initially, four (9.5%) samples tested positive for SARS‐CoV‐2 in the four locations that hosted COVID‐19 isolation facilities. Between February and October 2022, 345 specimens of wastewater were tested, and 99 (28.7%) were positive. The highest detection rate was in February, June, and July (60%, 45%, and 43%, respectively), which corresponded to COVID‐19 peaks during 2022, and the lowest detection rate was in August and September (11% and 0%, respectively), corresponding to the low number of COVID‐19 cases.

**Conclusion:**

The detection rate of SARS‐CoV‐2 in wastewater samples from Bahrain was high and was significantly correlated with the number of reported COVID‐19 cases. Wastewater surveillance can aid the existing surveillance system in monitoring SARS‐CoV‐2 spread.

## 1 INTRODUCTION

In March 2020, the World Health Organization declared COVID‐19 a pandemic.^1^ Since then, over 750 million cases have been detected, with more than six million deaths confirmed, making it one of the most extensive pandemics in history.^2^ COVID‐19 is caused by severe acute respiratory syndrome coronavirus 2 (SARS‐CoV‐2). Coronaviruses are single‐stranded RNA viruses belonging to the Coronavirdiae family that had two previously known highly pathogenic viruses, severe acute respiratory syndrome coronavirus (SARS‐CoV‐1) and Middle East respiratory coronavirus (MERS‐CoV), both cause severe respiratory diseases in humans.^3^

SARS‐CoV‐2 virus is a highly contagious virus that is stable in the environment and can mutate quickly. It is transmitted mainly through the respiratory route, with an evidence that it can act like enteric viruses and spread via the fecal–oral route.^4^, ^5^, ^6^ The possibility of transmission via other routes depends on the virus load and the surrounding environments.^6^ About half of the patients infected with COVID‐19 developed gastrointestinal symptoms with a detectable SARS‐CoV‐2 in their fecal samples.^3^, ^5^, ^7^, ^8^, ^9^ This was similar to SARS‐CoV‐1 and MERS‐CoV, which have gastrointestinal related symptoms.^10^, ^11^

SARS‐CoV‐2 was detected in the fecal specimen of the COVID‐19 patients.^8^ This indicates that the SARS‐CoV‐2 can be excreted through the infected individuals' feces or other body fluids and transported to the wastewater. Several studies have detected SARS‐CoV‐2 virus in wastewater.^12^, ^13^, ^14^ Due to the rate of transmissibility of SARS‐CoV‐2, early detection of the outbreak is crucial in controlling the spread of the disease. The diagnostic tests used to diagnose patients were not meant for mass surveillance as they are time consuming and costly.^15^ On the other hand, environmental surveillance of SARS‐CoV‐2 could be used as a data source, indicating the spread of the virus in the human population.^16^ Surveillance of wastewater can be used as a supplementary method to improve the monitoring of the disease spread and estimation of the prevalence of the disease in the community.^17^, ^18^ Wastewater surveillance is a method previously used to evaluate the spread of other viruses, such as norovirus, poliovirus, and measles viruses in communities.^6^

The Kingdom of Bahrain initiated wastewater surveillance for SARS‐CoV‐2 late in 2020 to aid in the testing strategies of the population and to monitor the disease activity in the community. This is done to overcome missing any asymptomatic cases that can pass unnoticed, resulting in an underestimation of COVID‐19 cases.^19^ Bahrain was following the test, trace, and treat strategy in combating COVID‐19 and had done one million polymerase chain reaction (PCR) tests in the first 5 months of the pandemic.^20^ The average number of PCR tests for COVID‐19 was between 10,000 and 20,000 PCR tests per day.

The aim of this study was to determine the presence of SARS‐CoV‐2 RNA in untreated wastewater samples in the Kingdom of Bahrain and to show the correlation between the presence of the virus in the wastewater and the number of diagnosed COVID cases in the same residential areas.

## 2 METHODS

### 2.1 Study design and study setting

A cross‐sectional retrospective design was adopted to review all the wastewater samples tested for SARS‐CoV‐2 in the public health laboratory, Ministry of Health, Manama, Bahrain between November 2020 and October 2022.

### 2.2 Sampling

Samples were collected from different residential blocks to cover all the governorates in the Kingdom of Bahrain using the grab method. Bahrain is a small country with 778 km^2^. It is divided into four main governorates and each governorate is subdivided into residential blocks.^21^ On average, 10 samples were collected weekly and covered all the four governorates. A total of 387 samples from different locations were collected and analyzed, 42 samples during November and December 2020 and 345 samples during February and October 2022. The samples were collected from a shared wastewater/sewage pipe of the residential block. The wastewater samples were collected in appropriate containers to provide enough sample volume for testing. The amount of raw sewage collected from the selected sampling sites was 1 L per sample. Samples were kept cold (<8°C) during transportation to the laboratory for processing. The exterior surfaces of sample containers were decontaminated with 70% isopropanol and incubated in a 60 ± 1°C water bath for 1.5 h, mixed once during incubation. The water level covered the containers to reach the target temperature. Samples were cooled to 2–8°C before proceeding with concentration. The pasteurized sample was stored overnight at 2–8°C.

### 2.3 Protocol for detection of SARS‐CoV‐2 from wastewater

The sample analysis method was adopted from the IDEXX reference laboratories kit in SARS‐CoV‐2 detection from sewage water which is a quantitative (RT‐qPCR) test that can detect and quantify RNA from the SARS‐CoV‐2 virus in untreated wastewater.^22^ We followed the protocol for preparation and concentration of wastewater samples prior to nucleic acid purification and quantification of SARS‐CoV‐2 using IDEXX Laboratories method too.^23^

### 2.4 Waste water sample concentration

The sample was mixed well; then, 35 ± 1 mL was added to three empty 50 mL centrifuge tubes and centrifuged at 4700 relative centrifugal force (RCF) for 30 min at 4 ± 1°C using a swinging bucket rotor centrifuge to provide a stable bacterial pellet. Tubes were removed gently from the centrifuge bucket, and supernatant from each tube was decanted carefully into three 50 mL tubes containing 3.5 ± 0.1 g polyethylene glycol (PEG) 8000 and 0.788 ± 0.01 g sodium chloride (NaCl) and mixed at ambient temperature until completely dissolved. The liquid was decanted from each tube into three new 50 mL centrifuge tubes and centrifuged at 12,000 RCF for 120 min at 4 ± 1°C. Then, the tubes were removed from the centrifuge, and most of the supernatant was discarded from each tube carefully to avoid interfering with viral pellets. The tubes were centrifuged at 12,000 RCF for 5 min at 4 ± 1°C, then removed carefully, and the remaining supernatant from each tube was discarded; 0.4 mL nuclease‐free water was transferred using pipettes to one of the tubes containing a viral pellet. The pellet was resuspended by repeatedly pipetting to rinse the inside surface of the tube around the pellet. The tube was flashed spin at 2000 ± 1000 RCF to collect all the liquid at the bottom of the tube, and then, the entire volume was transferred to the second tube containing the viral pellet after pipetting up and down several times to homogenize the concentrate. The same steps were repeated to transfer the entire volume to the third tube containing the viral pellet. Approximately 0.4 mL of the recovered concentrates were transferred to an RNase‐free microtube and proceeded with nucleic acid extraction, or it could be stored overnight at −25 to −15°C.

### 2.5 Real‐time PCR

One milliliter of pf PCR‐grade water was added to the SARS‐CoV‐2 mix (protected from light). Then, 200 μL of PCR‐grade water was added to the positive control and incubated at 18–26°C for 10 min (both tubes), Vortex, and centrifuge (both tubes). For each sample, 10 μL SARS‐CoV‐2 mix and 10 μL RNA master mix were added, accounting for 10% pipette loss. The SARS‐CoV‐2 mix was added to empty sterile Eppendorf tubes; then, RNA master mix was added and slowly pipetted to mix. The PCR mix was vortexed, and 20 μL was added to one well per sample plus four additional wells (negative and positive PCR control and RNA extraction negative and positive control). In the first well, 5 μL of sample (purified RNA) was added; in the second well, 5 μL of positive control was added, and 5 μL of PCR‐grade water (negative control) was added in the third well. Additional controls were prepared, and the plate was covered. The plate was then loaded into RT‐PCR, and the cycling program was initiated, and finally, the results were analyzed and reported as positive with cycle threshold (CT) value or negatives if the CT was 38 or more.

### 2.6 Data collection and analysis

The data collected include wastewater sample PCR results that were performed in public health laboratory in the Ministry of Health, samples identification number, date of sample collection, sample location (residential block and governorate), wastewater PCR results were reported as positive or negative, and CT value of positive results. The number of COVID‐19 cases was reported by residential blocks, governorates, and date of testing positive. All data were entered into Microsoft Excel for analysis. Descriptive analysis of quantitative variables was done using percentages, ratios, mean, and standard deviation. For correlation tests, IBM Statistical Package for the Social Sciences (SPSS) software version 26 for statistical analysis was used. Correlation between the rate of SARS‐CoV‐2 positive wastewater sample detection and the reported number of COVID‐19 cases in the same period was evaluated using Spearman's correlation test.

Similarly, testing for correlation between CT values of positive wastewater samples and the number of COVID‐19 cases in the same area and period was done using Spearman's correlation test. *P* values below 0.05 were considered statistically significant.

## 3 RESULTS

### 3.1 Detection of SARS‐CoV‐2 in wastewater samples

Of 387 wastewater samples collected, 103 (26.6%) samples tested positive for SARS‐CoV‐2. In the period between November and December 2020, a total of 42 samples were collected from wastewater drainage systems of 42 distinct residential blocks. Four (9.5%) samples tested positive for SARS‐CoV‐2. The positive samples were from four areas hosting COVID‐19 isolation and treatment facilities (Bahrain International Hospital, Sitra Isolation Center, Block 742 Aali, and Block 901 Hunayniyah).

Between February and October 2022 (corresponding to Epidemiological Weeks 5 to 44), 345 specimens of wastewater were collected from all four governorates of Bahrain: 103 (30%) in the Capital governorate, 90 (26%) in Muharraq governorate, 74 (21%) in the Northern governorate, and 78 (23%) in the Southern governorate. The samples were taken from 123 distinct residential blocks. Of the 345 specimens, 99 (29%) were positive for SARS‐CoV‐2 with an average CT value of 34.3. The distribution of positive samples was 41 (41%) in the Capital governorate, 20 (20%) in Muharraq governorate, 20 (20%) in the Northern governorate, and 18 (18%) in the Southern governorate (Table 1).

**TABLE 1:** Wastewater sample tested for SARS‐CoV‐2 by governorate in 2022 in Bahrain.

Governorate/sample results	Positive samples		Total	
	*N*	%	*N*	%
Capital governorate	41	42	103	30
Muharraq governorate	20	20	90	26
Northern governorate	20	20	74	21
Southern governorate	18	18	78	23
Total	99	100	345	100

### 3.2 Correlation between the presence of the virus in the wastewater and the number of reported COVID‐19 cases

**FIGURE 1:**
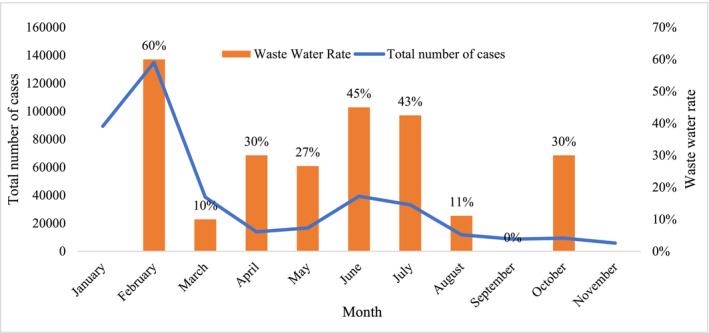
Rate of positive SARS‐CoV‐2 wastewater samples compared with the reported number of COVID‐19 cases by month of the year 2022 in the Kingdom of Bahrain.

Through 2022, the detection rate of positive wastewater samples was highest in February, June, and July, corresponding to 60%, 45%, and 43%, respectively. Those two periods corresponded to the two COVID‐19 peaks during the year: the first peak reached a record‐high number of cases in February (134,881 cases) and the second peak in June with cases reached 39,316. In April and May, the number of reported COVID‐19 cases had reduced to 13,947 and 16,686 cases, respectively, but the rate of positive sewage samples was noticeable, with both months recording 30% and 27%, respectively. The lowest wastewater detection rate was 11% and 0% in August and September, respectively. This period corresponded to a low number of positive COVID‐19 cases, with an average of 9956 ± 1360 cases per month. October showed a similar trend, with 9371 reported COVID‐19 cases but wastewater SARS‐CoV‐2 detection rate increased to 30% (Figure 1 and Table S1).

The data were then analyzed according to the epidemiological weeks. In 2022, 31 weeks were covered by wastewater surveillance. The number of wastewater samples tested per week ranged between 5 and 20 samples, with an average of 11 samples per week. The positivity rate was about 27% ± 22%.

Starting from Week 5 and through Weeks 6 and 8, the detection rate increased steadily from 50% to 60% to 70%, respectively. This period was consistent with the highest wave of COVID‐19 cases in the Kingdom of Bahrain, caused by the emerging Omicron variant of SARS‐CoV‐2. During this period, cases started to increase by Week 1 (7614 cases), reaching the highest peak in Week 5 (50,274 cases), then started to decline, reaching 20,510 cases in Week 8.

In Weeks 11, 12, and 13, positive sample detection rates were low, at around 10%. COVID‐19 cases during the same period continued to decline. Samples taken on Weeks 14 through 19 showed a higher detection rate of SARS‐CoV‐2, reaching 60% in Weeks 16 and 19. During this period, the number of COVID‐19 cases reported was stable, averaging around 3500 cases from Week 14 till Week 19.

Similar to the COVID‐19 wave in Weeks 5 to 8, the detection rate of positive wastewater samples increased progressively from Week 21 to Week 26, from 10% in Week 21 and reaching 75% in Week 26. Afterward, the ratio declined to reach 10% in Weeks 30, 31, and 32. COVID‐19 cases in the same period also showed a similar pattern, with cases started to increase in Week 21 (3181 cases), reaching a peak in Week 25 (12,334 cases), then started to decline to return to baseline in Week 31 (3131 cases).

**FIGURE 2:**
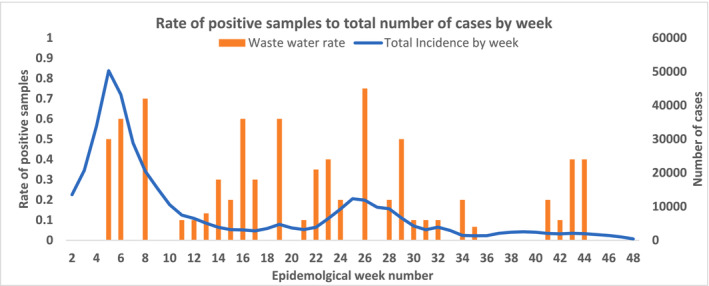
Rate of positive SARS‐CoV‐2 wastewater samples compared with the reported number of COVID‐19 cases by epidemiological weeks of the year 2022 in the Kingdom of Bahrain.

The reported number of COVID‐19 cases was mostly stable from Week 33 to Week 38; with an average of about 1900 cases per week. During this period, positive wastewater sample detection rate was the lowest, with Weeks 33, 36, 37, and 38 recording zero‐detection rate from a total of 40 samples collected. Although the number of reported COVID‐19 cases was also relatively stable from Week 41 till Week 44, with 2000 cases on average per week, there was a higher detection rate of positive wastewater samples, reaching 40% in Weeks 43 and 44 (Figure 2 and Table S2).

### 3.3 Correlation analysis

The rate of detection of SARS‐CoV‐2 in wastewater samples was compared with the number of COVID‐19 cases in the same epidemiological week, the number of cases the week before, and the week after using Spearman's correlation test. The rate of positive samples was significantly correlated with the reported number of COVID‐19 cases. A stronger correlation was found with the reported number of cases of the same epidemiological week (*r* = 0.5), followed by the number of cases reported in the week after (*r* = 0.479) and the number of cases reported in the week before (*r* = 0.434).

Detected CT values were compared to the reported COVID‐19 cases within the same residential blocks from where sewage samples were collected using Spearman's correlation test. The CT values detected from the 99 positive samples ranged from 30 to 37, the average CT value being 34 ± 1.6. We found a significant negative correlation of −0.45 between the CT values and COVID‐19 case counts. Lower CT values were correlated with a higher number of cases in the residential blocks from which samples were collected.

## 4 DISCUSSION

During the active phase of COVID‐19, the testing of samples was largely prompted by symptoms. People could have been shedding the virus before being symptomatic or may be asymptomatic, resulting in underdetection of the disease spread. This raises the need for a method that can help in a real‐time estimation of the extent of the disease within the community to help in modulating and adjusting the pandemic response promptly.^24^

The wastewater‐based surveillance provided an almost real‐time, low‐cost method to monitor the level of community transmission of SARS‐CoV‐2. The usefulness of wastewater surveillance had been limited by the difficulty in linking the prevalence of the virus in wastewater to the number of COVID‐19 positives in the community.^25^ In the current study, the level of detection of COVID‐19 in wastewater was correlated to the number of positive cases in the same epidemiological weeks. The correlation found between the presence of SARS‐CoV‐2 in wastewater and the reported number of COVID‐19 cases was consistent with results from regional studies, including studies conducted in Qatar and the UAE. In Qatar, it was observed that the measured overall SARS‐CoV‐2 declined in wastewater with the reduction in the number of daily reported cases and an upsurge in these reported cases corresponded with an increase in the viral load detected in wastewater.^26^ Similarly, in the UAE, the viral load detected in wastewater diminished during the period when government‐imposed measures led to a reduction in the number of recorded cases. As the daily reported cases began to increase, the viral loads detected correspondingly surged.^27^ The wastewater surveillance could serve as a data source and an indicator of the virus circulating within the population.^16^, ^28^

The level of SARS‐CoV‐2 can be an indication of the presence of a cluster of cases in certain areas and can help in detecting local outbreaks. This can assist public health and hospital officials in planning for the mitigation process as well as gauge hospital bed availability and the required care needed.^29^ The level of SARS‐CoV‐2 in wastewater is variable over time, and a peak of SARS‐CoV‐2 in wastewater increased prior to the increase in hospitalization.^24^ The wastewater surveillance in Bahrain was used as guidance for the COVID‐19 mobile and random testing unit which was directed to the area with positive wastewater samples to help in detecting local outbreaks. In this study, no correlation was made between the increase in hospitalization and the wastewater detection of the SARS‐CoV‐2.

In the current study, COVID‐19 cases were at par with the percentage of positive SARS‐CoV‐2 in wastewater during January and February 2022. Testing strategy during the pandemic included symptomatic people, asymptomatic contacts, travelers arriving in the country, and random testing done across the country for workers and in public places.^30^ The testing strategy was changed during March and April when travelers' testing was stopped, and testing was limited to symptomatic patients and symptomatic contacts.^28^ The change in the testing strategy affected the number of COVID‐19‐positive cases detected. Reducing the number of tests to detect COVID‐19 cases may result in underestimating the true number of infections. The change in testing strategies was undertaken in most countries during the pandemic, and the reduction in COVID‐19 test number may have resulted in a lower number of reported cases, thus not reflecting the actual degree of the spread of the infection.^31^ The detection of SARS‐CoV‐2 in wastewater can be affected by the location, sewer shed, population size, sampling strategies, and other factors like temperature, which make the wastewater a heterogeneous environment. SARS‐CoV‐2 was detected in some patients even before COVID‐19 symptoms which could explain the inconsistency between the detection of the virus and COVID‐related hospitalization.^32^

This study had some limitations. There is inconsistency in the blocks where the samples were collected. The samples were not collected from the same block each week, making following the trends of the spread of the disease difficult, but in most cases, samples were collected from the same governorate. This limitation was overcome by clumping the data of the blocks into the governorates. As Bahrain is a small country, presenting the data by governorate might be the best way of stratification and analysis.

Moreover, the wastewater surveillance was initiated in late 2020. However, it was not conducted regularly till 2022, making it difficult to correlate all the periods where there were surges in the number of COVID‐19 cases to the rate of wastewater detection. Despite these limitations, testing wastewater for COVID‐19 acted as an indicator of the spread of the disease in the community. This study showed a correlation between SARS‐CoV‐2 presence in wastewater and the number of COVID cases in the community. Wastewater surveillance for SARS‐CoV‐2 can be used with limited resources instead of mass testing or as an adjuvant to help map the spread of the outbreak or monitor the spread. It can also be used to monitor disease activity in the long term beyond the pandemic to keep an eye on the emergence of new virus variants.

## 5 CONCLUSIONS

In summary, the 2020 initial testing period showed that the highest detection rates of COVID‐19 in wastewater samples were found in areas where COVID‐19 isolation and treatment facilities were hosted. In the 2022 testing period, a higher detection rate was significantly correlated with the number of reported COVID‐19 cases. During the peaks of reported COVID‐19 cases, there were similar peaks in the number of positive wastewater samples. However, some peaks of positive wastewater samples were not accompanied by a corresponding peak in reported COVID‐19 cases. Similar trends were observed when comparing the percentage of positive wastewater samples and cases per epi‐week. These findings emphasize the importance of wastewater surveillance, which could be used more efficiently in future pandemics. Combined with other testing strategies, it offers a wider picture of the spread of the disease in a community compared to individual testing only. Additionally, wastewater surveillance offers valuable data guiding policymakers and helping to monitor the effectiveness of public health measures implemented.

## AUTHOR CONTRIBUTIONS

**Afaf Merza Mohamed:** Conceptualization; formal analysis; project administration; methodology; supervision; validation; writing—original draft; writing—review and editing. **Ebrahim Matar:** Formal analysis; software; validation; writing—original draft; writing—review and editing. **Hasan M. Isa:** Validation; supervision; writing—original draft; writing—review and editing. **Ahmed K. Moosa:** Writing—original draft; writing—review and editing. **Wafa Fawzi Hasan:** Formal analysis; software; validation; writing—original draft; writing—review and editing. **Amjad Ghanem Mohamed:** Conceptualization; data curation; formal analysis; investigation; project administration; validation; supervision; writing—review and editing. **Adel Salman Al Sayyad:** Conceptualization; formal analysis; methodology; validation; supervision; writing—review and editing. **Maryam Y. Sanad:** Data curation; investigation; writing—original draft. **Maryam Alhajeri:** Conceptualization. **Najat Abu Alfatah:** Conceptualization. **Qasim M. Alaraibi:** Data curation; investigation.

## CONFLICT OF INTEREST STATEMENT

No financial or non‐financial benefits have been received or will be received from any party directly or indirectly related to this article's subject.

### PEER REVIEW

The peer review history for this article is available at https://www.webofscience.com/api/gateway/wos/peer-review/10.1111/irv.13194.

## ETHICS STATEMENT

This study was conducted in accordance with the principles of Helsinki Declaration, and it was ethically approved by the Health Research Committee in the Ministry of Health, Kingdom of Bahrain.

## DISCLAIMERS

The views expressed in the submitted article are our own and not an official position of any institution or funder.

## CONSENT FORM

Data from laboratory samples and COVID‐19 data were statistically analyzed without any patient's identification number.

## Supporting information


**Table S1:** Rate of positive SARS‐CoV‐2 wastewater samples in relation to the number of COVID cases, COVID test, and rate of positive tests by month in Bahrain in 2022.

**Table S2:** Rate of positive SARS‐CoV‐2 wastewater samples, number of new COVID‐19 cases, number of COVID‐19 tests done, rate of positive COVID‐19 tests by months of 2022, in the Kingdom of Bahrain.

## Data Availability

The data that support the findings of this study are available from the corresponding author upon reasonable request.
