# Supplementary material for: Presence of SARS‐CoV‐2 virus in wastewater in the Kingdom of Bahrain during the COVID‐19 pandemic
Source: Influenza Other Respir Viruses. 2023 Nov 13;17(11):e13194. doi: 10.1111/irv.13194 (PMC10642395; doi:10.1111/irv.13194)
Supplement: Supplementary file 1 — Table S1: Rate of positive SARS‐CoV‐2 wastewater samples in relation to the number of COVID cases, COVID test, and rate of positive tests by month in Bahrain in 2022. [file IRV-17-e13194-s001.docx]

| **Supplementary Table 1: Rate of positive SARS-CoV-2 wastewater samples in relation to the number of COVID cases, COVID test, and rate of positive test by month in Bahrain in 2022** | | | | |
| --- | --- | --- | --- | --- |
| **Month** | **Waste Water Rate** | **Total number of cases** | **Monthly Tests** | **COVID 19 Positivity Rate** |
| **January** | -- | 89338 | 752584 | 12% |
| **February** | 60% | 134881 | 616687 | 22% |
| **March** | 10% | 38673 | 211474 | 18% |
| **April** | 30% | 13947 | 112947 | 12% |
| **May** | 27% | 16686 | 128365 | 13% |
| **June** | 45% | 39316 | 173415 | 23% |
| **July** | 43% | 33232 | 147207 | 23% |
| **August** | 11% | 11836 | 107251 | 11% |
| **September** | 0% | 8662 | 102059 | 8% |
| **October** | 30% | 9371 | 111121 | 8% |
| **November** | -- | 5872 | 81384 | 7% |
