# Supplementary material for: Presence of SARS‐CoV‐2 virus in wastewater in the Kingdom of Bahrain during the COVID‐19 pandemic
Source: Influenza Other Respir Viruses. 2023 Nov 13;17(11):e13194. doi: 10.1111/irv.13194 (PMC10642395; doi:10.1111/irv.13194)
Supplement: Supplementary file 2 — Table S2: Rate of positive SARS‐CoV‐2 wastewater samples, number of new COVID‐19 cases, number of COVID‐19 tests done, rate of positive COVID‐19 tests by months of 2022, in the Kingdom of Bahrain. [file IRV-17-e13194-s002.docx]

| **Supplementary Table 2: Rate of positive SARS-CoV-2 wastewater samples, number of new COVID-19 cases, number of COVID-19 tests done, rate of positive COVID-19 tests by months of 2022, in the Kingdom of Bahrain.** | | | | | | |
| --- | --- | --- | --- | --- | --- | --- |
| **Week number** | **Number of tests** | **Wastewater rate** | **Incidence in the week before** | **Incidence at the same week** | **Incidence in the week after** | **Total incidence of 3 weeks** |
| Week 1 | - | - | - | 0 | 0 | - |
| Week 2 | - | - | 7614 | 13536 | 20712 | 41862 |
| Week 3 | - | - | 13536 | 20712 | 33844 | 68092 |
| Week 4 | - | - | 20712 | 33844 | 50274 | 104830 |
| Week 5 | 10 | 50% | 33844 | 50274 | 43198 | 127316 |
| Week 6 | 20 | 60% | 50274 | 43198 | 28837 | 122309 |
| Week 7 | - | - | 43198 | 28837 | 20510 | 92545 |
| Week 8 | 10 | 70% | 28837 | 20510 | 15387 | 64734 |
| Week 9 | - | - | 20510 | 15387 | 10558 | 46455 |
| Week 10 | - | - | 15387 | 10558 | 7466 | 33411 |
| Week 11 | 10 | 10% | 10558 | 7466 | 6475 | 24499 |
| Week 12 | 10 | 10% | 7466 | 6475 | 5070 | 19011 |
| Week 13 | 15 | 13% | 6475 | 5070 | 3837 | 15382 |
| Week 14 | 10 | 30% | 5070 | 3837 | 3152 | 12059 |
| Week 15 | 10 | 20% | 3837 | 3152 | 3080 | 10069 |
| Week 16 | 5 | 60% | 3152 | 3080 | 2784 | 9016 |
| Week 17 | 10 | 30% | 3080 | 2784 | 3494 | 9358 |
| Week 18 | - | - | 2784 | 3494 | 4726 | 11004 |
| Week 19 | 10 | 60% | 3494 | 4726 | 3680 | 11900 |
| Week 20 | - | - | 4726 | 3680 | 3181 | 11587 |
| Week 21 | 10 | 10% | 3680 | 3181 | 3865 | 10726 |
| Week 22 | 20 | 35% | 3181 | 3865 | 6430 | 13476 |
| Week 23 | 10 | 40% | 3865 | 6430 | 9258 | 19553 |
| Week 24 | 10 | 20% | 6430 | 9258 | 12334 | 28022 |
| Week 25 | - | - | 9258 | 12334 | 11886 | 33478 |
| Week 26 | 20 | 75% | 12334 | 11886 | 9802 | 34022 |
| Week 27 | - | - | 11886 | 9802 | 9344 | 31032 |
| Week 28 | 10 | 20% | 9802 | 9344 | 6645 | 25791 |
| Week 29 | 10 | 50% | 9344 | 6645 | 4253 | 20242 |
| Week 30 | 10 | 10% | 6645 | 4253 | 3131 | 14029 |
| Week 31 | 10 | 10% | 4253 | 3131 | 3896 | 11280 |
| Week 32 | 10 | 10% | 3131 | 3896 | 2925 | 9952 |
| Week 33 | 10 | 0% | 3896 | 2925 | 1438 | 8259 |
| Week 34 | 10 | 20% | 2925 | 1438 | 1342 | 5705 |
| Week 35 | 15 | 7% | 1438 | 1342 | 1367 | 4147 |
| Week 36 | 10 | 0% | 1342 | 1367 | 2081 | 4790 |
| Week 37 | 10 | 0% | 1367 | 2081 | 2408 | 5856 |
| Week 38 | 10 | 0% | 2081 | 2408 | 2535 | 7024 |
| Week 39 | - | - | 2408 | 2535 | 2402 | 7345 |
| Week 40 | - | - | 2535 | 2402 | 2043 | 6980 |
| Week 41 | 5 | 20% | 2402 | 2043 | 1911 | 6356 |
| Week 42 | 10 | 10% | 2043 | 1911 | 2093 | 6047 |
| Week 43 | 15 | 40% | 1911 | 2093 | 1970 | 5974 |
| Week 44 | 10 | 40% | 2093 | 1970 | 1688 | 5751 |
| Week 45 | - | - | 1970 | 1688 | 1421 | 5079 |
| Week 46 | - | - | 1688 | 1421 | 1009 | 4118 |
| Week 47 | - | - | 1421 | 1009 | 417 | 2847 |
